# Fungal lectin MpL enables entry of protein drugs into cancer cells and their subcellular targeting

**DOI:** 10.18632/oncotarget.15849

**Published:** 2017-03-02

**Authors:** Simon Žurga, Milica Perišić Nanut, Janko Kos, Jerica Sabotič

**Affiliations:** ^1^ Department of Biotechnology, Jožef Stefan Institute, Ljubljana, Slovenia; ^2^ University of Ljubljana, Faculty of Pharmacy, Ljubljana, Slovenia

**Keywords:** lectin, drug delivery, cancer invasion, peptidase inhibitor, fusion proteins

## Abstract

Lectins have been recognized as promising carrier molecules for targeted drug delivery. They specifically bind carbohydrate moieties on cell membranes and trigger cell internalization. Fungal lectin MpL (*Macrolepiota procera* lectin) does not provoke cancer cell cytotoxicity but is able to bind aminopeptidase N (CD13) and integrin α3β1, two glycoproteins that are overexpressed on the membrane of tumor cells. Upon binding, MpL is endocytosed in a clathrin-dependent manner and accumulates initially in the Golgi apparatus and, finally, in the lysosomes. For effective binding and internalization a functional binding site on the α-repeat is needed. To test the potential of MpL as a carrier for delivering protein drugs to cancer cells we constructed fusion proteins consisting of MpL and the cysteine peptidase inhibitors cystatin C and clitocypin. The fused proteins followed the same endocytic route as the unlinked MpL. Peptidase inhibitor-MpL fusions impaired both the intracellular degradation of extracellular matrix and the invasiveness of cancer cells. MpL is thus shown *in vitro* to be a lectin that can enable protein drugs to enter cancer cells, enhance their internalization and sort them to lysosomes and the Golgi apparatus.

## INTRODUCTION

Lectins are a structurally diverse group of proteins that specifically and reversibly bind to carbohydrates. They act as recognition and adhesion molecules and as signal transducers involved in a plethora of physiological functions [1, 2]. They possess at least one carbohydrate binding domain which, when these domains are combined, enables their multivalency and cross-linking of glycoconjugates [1, 3]. The first discovered and the most extensively investigated are lectins from plants, however, in recent years fungal lectins have been gaining more attention [4, 5]. Fungal lectins differ from their counterparts from other species in their unique structures and binding specificities [6]. They have shown to be very stable proteins, resistant to changes in pH and temperature as well as to proteolytic digestion, which strengthens their potential for application in biotechnology and medicine. We have previously isolated and characterized biochemically a novel lectin from *Macrolepiota procera* designated as MpL [7]. MpL is structurally similar to the B subunit of ricin, a lectin from the castor bean *Ricinus communis*, a canonical member of the β-trefoil-type lectins [8, 9]. Fungal β-trefoil type lectins exhibit remarkable diversity in their binding partners, which include carbohydrates and proteins [6, 10, 11]. MpL is active as a dimer and possesses a strong carbohydrate binding domain specific for terminal N-acetyllactosamine and other β-galactosides on α-repeat and a putative carbohydrate binding domain with unknown specificity on γ-repeat [7].

Cell membrane proteins and lipids are specifically glycosylated and, as such, are potential binding sites for lectins, which could therefore be applied for targeted delivery of drugs. Since expression of carbohydrate structures is altered during the progression of cancer, the lectins distinguish between particular cell subsets and enable more precise recognition of cancer cells than do other ligands used in active drug delivery systems [2, 12, 13]. Besides recognizing specific glycosylation patterns on cancer cells, some lectins trigger internalization and can be used to deliver other molecules into the cells and to sort them to intracellular compartments. The B subunit of ricin, which enables its entry into cells through at least six different routes [14, 15], is currently being investigated as a protein carrier for lysosomal delivery in enzyme replacement therapy [15].

Lysosomal targets, including peptidases, constitute a challenge in cancer treatment. The invasiveness of cancer cells is known to depend on the degradation of extracellular matrix (ECM), a barrier that prevents cancer cells from migrating and invading distant sites. ECM is degraded extracellularly predominantly by matrix metallopeptidases, however, the process is greatly accelerated if fragments of ECM proteins are internalized by cancer cells and ultimately degraded within the lysosomes. We and others have shown that inhibition of lysosomal peptidases impairs cancer cell invasion [16–18]. Cathepsins, lysosomal cysteine peptidases, are particularly important in this process and can be targeted in the lysosomes by small inhibitors which can enter the cells by diffusion, or by specific protein inhibitors, such as clitocypin and cystatin C. Clitocypin is a fungal inhibitor of cysteine peptidases that shares the same β-trefoil fold as MpL and is an inhibitor of papain-like cysteine peptidases such as papain, cathepsins L, V, S and K, but a weak inhibitor of cathepsin B [19–21]. Human cystatin C is a potent inhibitor of papain-like cysteine peptidases, including cathepsin B [22–24]. However, the ability of protein inhibitors to enter the cells and localize within the lysosomes is very low [25]. Delivery systems able to guide peptidase inhibitors to lysosomes could significantly enhance cell uptake and increase inhibition of lysosomal proteolytic activity.

In this study we applied MpL, a recombinant lectin from *Macrolepiota procera*, for potential delivery of peptidase protein inhibitors to lysosomes. We evaluated the effects of MpL on different cancer cell lines, the binding partners on cell membrane, the mechanisms of internalization and its final destination within the cells. In order to test its potential as a carrier molecule in targeted delivery we constructed fusion proteins of MpL and peptidase inhibitors and investigated their cell uptake and effects on cancer cell invasiveness.

## RESULTS

### Effects of MpL on different human cell lines

Given that MpL has been reported to be toxic towards the model nematode *Caenorhabditis elegans* [7] we tested its effects on different human cell lines. In the viability loss assay MpL was shown to be non-toxic to any of the suspension cells (NK-92, Jurkat, non-differentiated U937 cells), and adherent cells (HeLa, HepG2, SH-SY5Y, MCF10A neoT and phorbol 12-myristate 13-acetate (PMA) differentiated U937 cells) at three different concentrations (0.2 μM, 1 μM and 5 μM) and at three different time points (48 h, 72 h and 96 h) (Table 1, Supplementary Tables 2 and 3).

**Table 1:** The viability of several human cell lines is unaffected by MpL

	Cell viability			
Cell line	Non-treated cells (viability - %)	Treated cells: 0.2 μM (relative viability - %)	Treated cells: 1 μM (relative viability - %)	Treated cells: 5 μM (relative viability - %)
HeLa	100.00 ± 1.85	105.60 ± 5.34	105.51 ± 0.65	107.26 ± 8.42
SH-SY5Y	100.00 ± 7.41	110.10 ± 9.09	101.45 ± 5.13	104.44 ± 9.76
HepG2	100.00 ± 16.65	90.29 ± 5.85	122.42 ± 12.81	92.58 ± 24.54
MCF10A neoT	100.00 ± 5.23	87.36 ± 10.74	116.28 ± 19.28	112.43 ± 22.06
U937	100.00 ± 2.27	97.78 ± 10.51	100.51 ± 2.80	113.78 ± 3.30
Differentiated U937	100.00 ± 5.74	111.97 ± 4.75	116.34 ± 4.26	119.78 ± 7.40
NK-92	100.00 ± 18.53	97.44 ± 6.73	68.11 ± 11.57	86.42 ± 9.37
Jurkat	100.00 ± 8.98	92.93 ± 8.23	101.70 ± 6.52	104.73 ± 4.81

Cell viability of different cell lines as measured by the MTS assay shows no significant toxicity of lectin MpL at three different concentrations (0.2 μM, 1 μM and 5 μM).

**Table 2:** Identification of MpL targets by mass fingerprinting

Protein (UniProt)	Identified protein (gene)	Glycoprotein	Cellular localization	No. of unique peptides		
				Sample 1	Sample 2	Sample 3
P15144	Aminopeptidase – CD13 (ANEP)	N- and O-glycosylated	Membrane		5	
P26006	Isoform α3A of integrin α3 – CD49c (ITGA3)	N-glycosylated	Membrane	4		
P05556	Isoform β1A of integrin β1 – CD29 (ITGB1)	N-glycosylated	Membrane	3		
P02751	Fibronectin (FN1)	N-glycosylated	Secreted	1		7
P25311	Zinc-alpha-2 glycoprotein (AZGP1)	N-glycosylated	Secreted			2

The potential MpL targets on MCF10A neoT cells were obtained by co-immunoprecipitation and mass spectrometry.

### MpL is rapidly internalized into human cell lines

**Figure 1:**
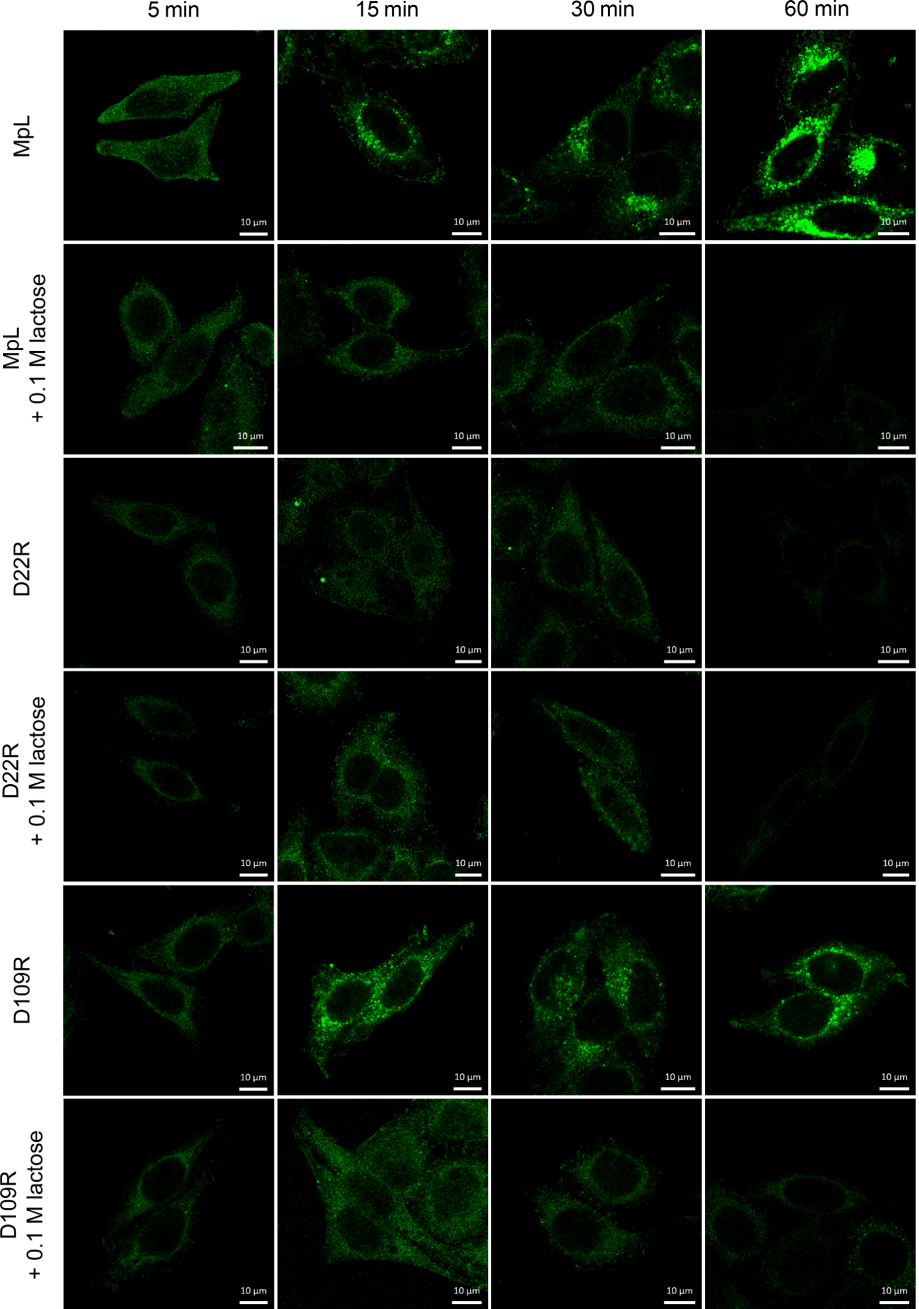
Localization of MpL and non-glycan-binding MpL mutants in HeLa cells Localization of MpL, D22R and D109R in HeLa cells, in the absence or presence of 0.1 M lactose at 5, 15, 30 and 60 min after addition of lectins. Cells were stained with anti-MpL antibodies and secondary goat anti-rabbit antibodies conjugated with Alexa Fluor 488. Images were taken at 63x magnification.

The binding of MpL to cell surface receptors is dependent on glycan binding as was shown by utilizing MpL mutants and inhibition assay by lactose (Figure 1, Supplementary Figure 1). MpL bound to the surface of HeLa and MCF10A neoT cells almost immediately after being added to the cell culture medium and, after 15 minutes, the lectin could already be observed inside the cells (Figure 1, Supplementary Figure 1). The D109R mutant, with an inactive binding site on the γ-repeat and a functional binding site on the α-repeat showed characteristics regarding cell entry similar to those of the wild-type MpL, indicating that the binding site on the α-repeat mediates its internalization. Indeed, the D22R mutant, with an inactive binding site on the α-repeat, did not enter the cells (Figure 1, Supplementary Figure 1). Similarly, when lactose (0.1 M), a competitive inhibitor of glycan binding to the MpL's α-repeat, was added to the medium, internalization of MpL and D109R was blocked. The addition of lactose did not, however, affect the binding of the D22R mutant (Figure 1, Supplementary Figure 1). Initial weak binding to the cell surface in the first 30 minutes after addition was observed for D22R and for all samples that contained lactose (Figure 1, Supplementary Figure 1), presumably as a result of non-specific binding. Similar binding and internalization of MpL was observed in other cell lines tested: PMA differentiated and non-differentiated U937, NK-92 and Jurkat cells (Supplementary Figure 2).

### Subcellular localization of MpL in human cell lines

**Figure 2:**
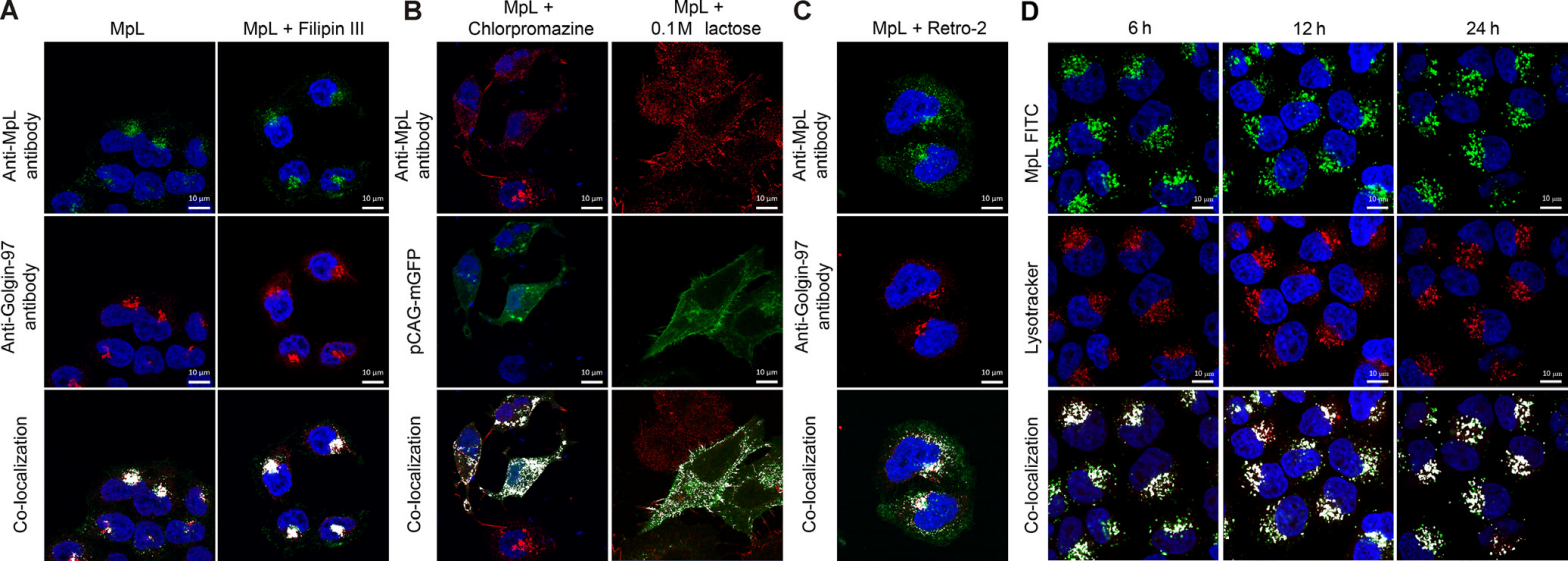
Mechanisms of MpL cell uptake and sorting (**A**) Localization of MpL in HeLa cells after 3 h incubation in the presence or absence of Filipin III. Cells were double labelled with anti-MpL antibodies and anti-human Golgin-97 antibodies and secondary antibodies conjugated with Alexa Fluor 488 (MpL) and Alexa Fluor 555 (Golgin-97). (**B**) Localization of MpL on the plasma membrane of HeLa cells after 3 h incubation in the presence of chlorpromazine or 0.1 M lactose. Cells were transfected with the pCAG-mGFP plasmid for membrane staining and stained with anti-MpL antibodies and secondary antibodies conjugated with Alexa Fluor 555. (**C**) Localization of MpL in HeLa cells after 3 h incubation in the presence of Retro-2. Cells were double labelled with anti-MpL antibodies and anti-human Golgin-97 antibodies and secondary antibodies conjugated with Alexa Fluor 488 (MpL) and Alexa Fluor 555 (Golgin-97). (**D**) Localization of FITC labelled MpL in LysoTracker Red DND 99 stained HeLa cells after 6, 12 and 24 h incubation. LysoTracker Red DND-99 was added to cell cultures 40 min prior to paraformaldehyde fixation. Images were taken at 63x magnification. Co-localization is shown in the merged pictures with white pixels.

Shortly after internalization, MpL could be observed in endosomal “dot like” structures and components of the Golgi apparatus (GA), as shown in HeLa and MCF10A neoT cells double-labelled with the Golgi apparatus marker (Golgin-97) and anti-MpL antibodies (Figure 2A, Supplementary Figure 3A).

MpL is internalized by clathrin-dependent endocytosis, as shown by employing endocytosis inhibitors. Inhibition of clathrin-independent endocytosis by Filipin III (the inhibitor of caveolae formation) had no effect on MpL uptake (Figure 2A, Supplementary Figure 3A). However, the inhibition of clathrin-mediated endocytosis by chlopromazine completely abolished the uptake of MpL into cells, being co-localized with the cell membrane labelled with embedded palmitoylated GFP (Figure 2B, Supplementary Figure 3B). Similar accumulation of membrane bound MpL was observed in the presence of lactose (Figure 2B, Supplementary Figure 3B). Furthermore, MpL co-localized with transferrin receptor, a well-established marker of the clathrin -dependent pathway (Supplementary Figure 4). Finally, the high rate of internalization of MpL further supports clathrin -mediated endocytosis, since it is considered to be a process around 100 fold faster than that of clathrin -independent, especially caveolae-dependent, endocytosis (t_1/2_ ≤ 20 min) [26].

The blockade of retrograde transport from early endosomes to Golgi apparatus by Retro-2 caused a decrease in the accumulation of MpL in the Golgi apparatus (Figure 2C, Supplementary Figure 3C). Staining the acidic sub-cellular compartments with LysoTracker Red DND-99 upon the blockade of retrograde transport with Retro-2 showed a significant co-localization with FITC labeled MpL after 3h incubation (Supplementary Figure 3E).

MpL was retained in endo/lysosomal compartments for an extended period of time (24 h) in both HeLa (Figure 2D) and MCF10A neoT cells (Supplementary Figure 3D).

### MpL binds to different glycosylated proteins on the cell surface membrane

Three glycosylated membrane proteins and two secreted glycosylated proteins were identified as targets of MpL on MCF10A neoT cells (Table 2). In order to determine specific MpL targets, the MCF10A neoT cell membrane fraction was isolated and incubated with MpL, resulting in complexes by co-immunoprecipitation. Comparison of the co-immunoprecipitates formed in the absence or presence of lactose revealed three major bands corresponding to target proteins of MpL on MCF10A neoT cells (Supplementary Figure 5). The best protein coverage was obtained through mass fingerprinting for aminopeptidase N (CD13), isoform α3A of integrin α3 (CD49c), and isoform β1A of integrin β1 (CD29). These three targets are cell membrane proteins with known glycosylation sites for the potential binding of MpL. Co-immunoprecipitation of aminopeptidase N, (CD13) and integrin β1 (CD29) with MpL was confirmed by western blot analysis (Supplementary Figure 5). Less well covered were secreted glycosylated proteins zinc-alpha-2 glycoprotein and fibronectin.

**Figure 3:**
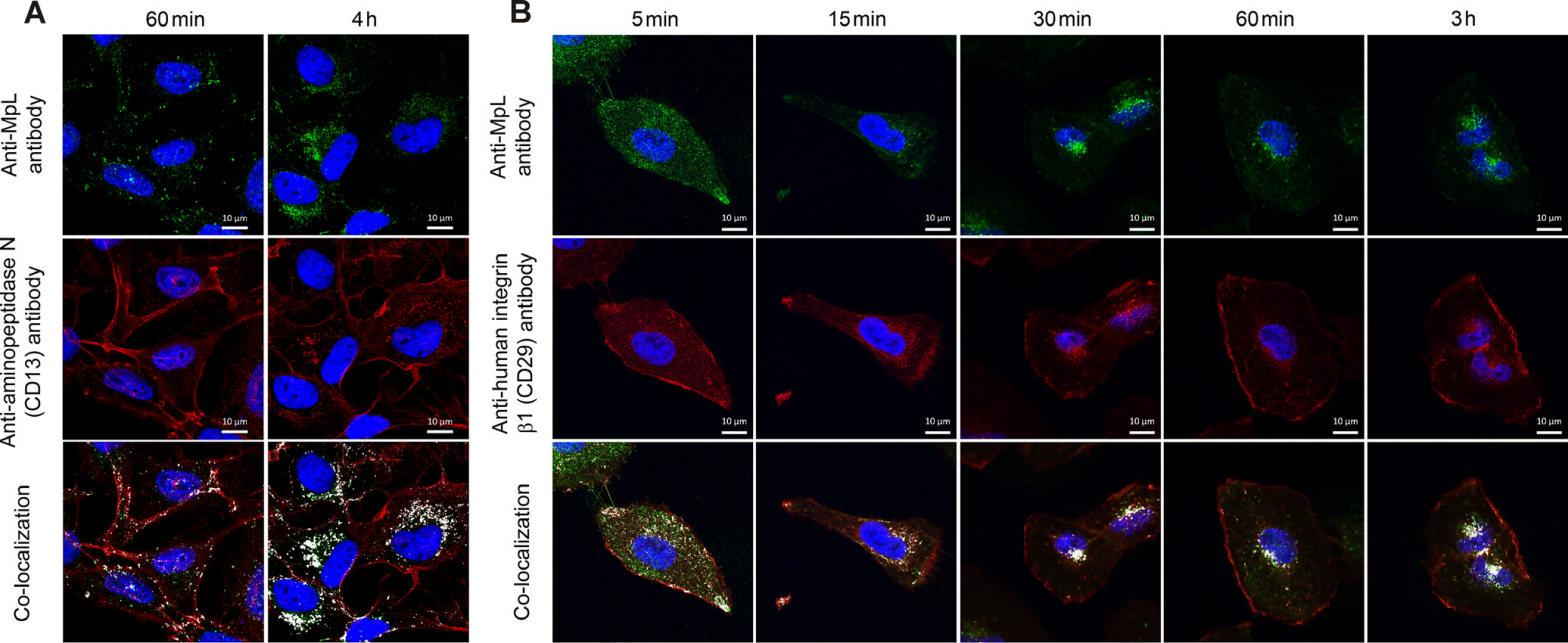
Co-localization of aminopeptidase N and integrin β1 and MpL in MCF10A neoT cells (**A**) Co-localization (at 60 min and 4 h) of MpL and aminopeptidase N (CD13) in MCF10A neoT cells. (**B**) Co-localization (at 5 min-3 h) and trafficking of MpL and integrin subunit β1 in MCF10A neoT cells. All cells were stained with rabbit anti-MpL antibodies and secondary goat anti-rabbit antibodies conjugated with Alexa Fluor 488 and mouse anti-human aminopeptidase N (CD13) or mouse anti-human integrin β1 (CD29) and secondary donkey anti-mouse antibodies conjugated with Alexa Fluor 555. Images were taken at 63x magnification. Co-localization is shown in the merged pictures with white pixels.

MpL and aminopeptidase N (CD13) co-localize in MCF10A neoT cells (Figure 3A). The co-localization was observed 60 min after addition of the lectin and increased with time, revealing a significant co-localization signal, mostly intracellularly. At the beginning of the experiment only a small portion of aminopeptidase N was detected intracellularly, indicating that MpL increased its internalization rate.

MpL co-localizes with the β1 integrin subunit in MCF10A neoT (Figure 3B) and HeLa cells (not shown) shortly after its addition to the cell culture medium. Antibody raised against the β1 subunit was used to analyse the subcellular distribution of α3β1 integrin as the β1 integrin subunit assembles with α3 integrin subunit to form heterodimeric receptors [27] and only these two integrin isoforms were identified by co-immunoprecipitation with MpL. Within a few minutes co-localisation occurred, presumably on the cell membrane whereas, after 15 min, both proteins could be seen intracellularly. After 30 min the extent of co-localization did not change and remained the same throughout the experiment (3 h). Part of the β1 integrin, however, remained on the cell's membrane.

### Effects of fusion proteins CysC-MpL and Clt-MpL on invasion of MCF10A neoT cells

**Figure 4:**
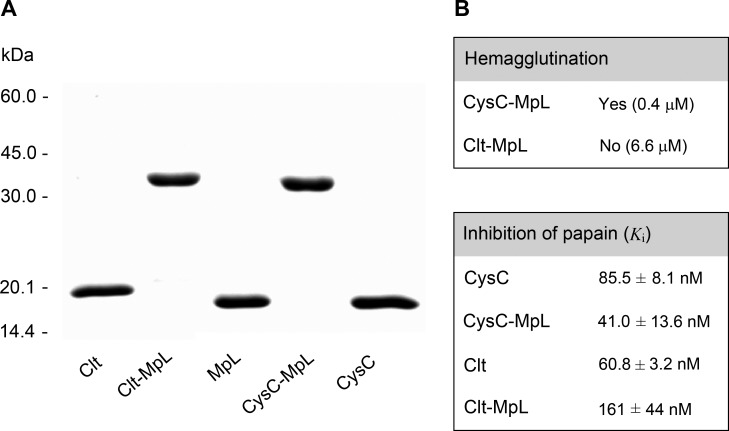
Purity and activity of fusion proteins (**A**) SDS-PAGE under nonreducing conditions. (**B**) Fusion protein activity as determined by hemagglutination assay and by measuring the inhibition of cysteine protease papain.

In order to explore the potential of MpL for delivering biological drugs to their intracellular targets we constructed two recombinant fusion proteins: MpL with each of the peptidase inhibitors clitocypin (Clt-MpL) and cystatin C (CysC-MpL). Clitocypin (Clt, Uniprot ID: Q3Y9I6) and cystatin C (CysC, UniProt ID: P01034) have both a low molecular weight – cystatin C of 13.4 kDa and clitocypin of 16.7 kDa – and are therefore suitable for constructing fusions. Furthermore, cystatin C is by 2 to 4 orders of magnitude more potent cysteine cathepsin inhibitor compared to clitocypin. Peptidase inhibitor (N-terminal part of fusion) was connected to the lectin domain (C-terminal part of fusion) with a long flexible peptide linker (GGGGS)_3_, forming the N-terminal region of fusion as described [28] and ensuring the retention of integrity/activity of both subunits. Both fusion proteins, Clt-MpL and CysC-MpL, were expressed in *E. coli* and purified (Figure 4A).

Both the lectin and peptidase inhibitor domains of fusions were active, as determined by haemagglutination assay and by measuring their inhibitory activity against the cysteine peptidase papain. CysC-MpL agglutinated human blood group B erythrocytes, whereas Clt-MpL did not, even at 6.6 μM (Figure 4B). However, immunocytochemical analysis, using anti-MpL specific antibodies, showed that Clt-MpL entered the subcellular compartments of HeLa (not shown) and MCF10A neoT cells (Supplementary Figure 6B), indicating that its lectin domain is active. CysC-MpL and Clt-MpL were active against papain with a constant of inhibition (*K*_i_) in the range of those determined for unlinked CysC and Clt (Figure 4B).

Both fusions, CysC-MpL and Clt-MpL, entered MCF10A neoT cells (Supplementary Figure 6) and HeLa cells (not shown) in a fashion similar to that of unlinked MpL. Shortly after addition, CysC-MpL (Supplementary Figure 6A) and Clt-MpL (Supplementary Figure 6B) entered the MCF10A neoT cells and co-localized with components of GA whereas, after prolonged incubation, they were present in the endo/lysosomal system. As for MpL, both fusions were retained in the endo/lysosomal compartments for an extended time period (Supplementary Figure 6).

Further, we tested the effects of CysC-MpL and Clt-MpL fusions on the activity of the intracellular peptidases cathepsin B and L (Supplementary Figure 7). Activity was measured in whole cell lysates prepared from cells treated with either fusion protein or with MpL, alone or in combination with each peptidase inhibitor, for one or two hours. The activity of cathepsin L was inhibited only in cells treated with CysC-MpL fusion (∼18% inhibition of activity compared to that of control). The activity of cathepsin B was reduced in cells treated with: CysC-MpL fusion (5.5% inhibition), a combination of unlinked CysC and MpL (∼ 10% inhibition), and a combination of unlinked Clt and MpL (11% inhibition). In the cell lysates treated with Clt and CysC alone no change in the activity of cathepsins B and L was detected.

**Figure 5:**
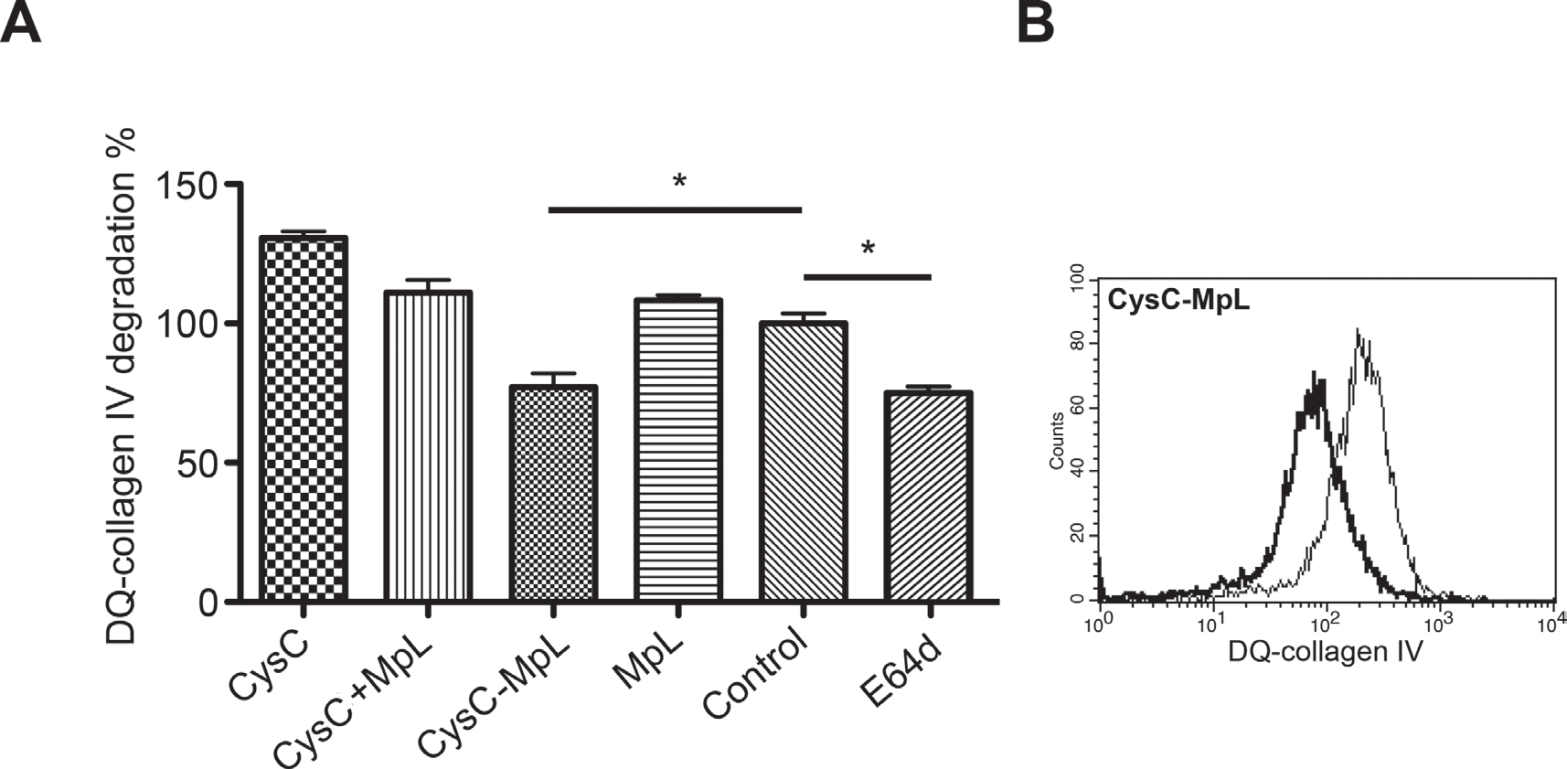
The fusion protein CysC-MpL inhibits intracellular DQ–collagen IV degradation by MCF10A neoT cells (**A**) Inhibition of intracellular DQ–collagen IV degradation by CysC-MpL as measured by flow cytometry. Flow cytometric data are presented as percentages of DQ–collagen IV degradation compared to the DMSO control. Error bars represent standard deviation of three replicates. Statistic indicator *P ≤ 0.05. (**B**) Inhibition of DQ–collagen IV degradation is represented by a shift in fluorescence intensity (thick line) as compared to the control MCF10A neoT cells treated with DMSO (thin line).

Reducing intracellular ECM degradation by internalized peptidase inhibitors may decrease the invasion of tumor cells through Matrigel. We analysed the intracellular degradation of ECM by following DQ-collagen IV in MCF10A neoT cells treated with fusion proteins, MpL alone or in combination with each peptidase inhibitor. Flow cytometric analysis showed a significant reduction of DQ-collagen IV degradation in the cells treated with CysC-MpL fusion, similar to the effect of intracellular protease inhibitor E64d (Figure 5) whereas Clt-MpL fusion was not effective (not shown).

**Figure 6:**
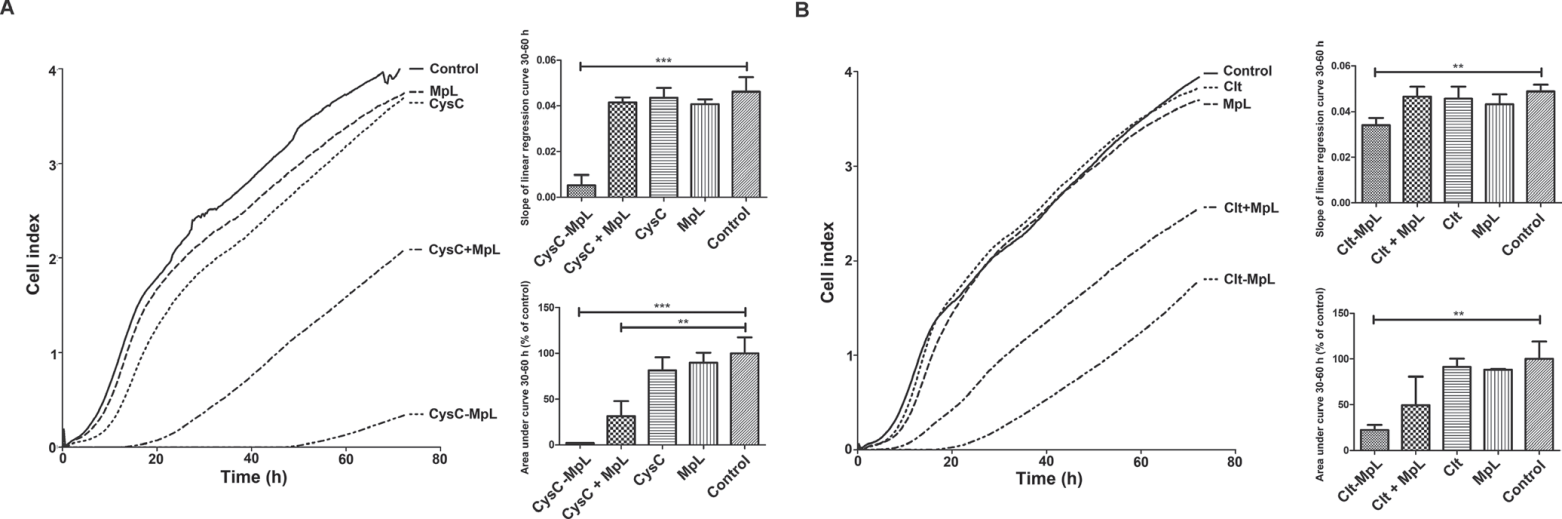
Inhibition of invasion of MCF10A neoT breast cancer cells through Matrigel coating by fusion proteins CysC-MpL and Clt-MpL Invasion of serum-starved MCF10A neoT cells was measured on a real-time cell analyser xCELLigence using CIM plates and Matrigel in the 72 hour time period. (**A** – CysC-MpL experiments; **B** – Clt-MpL experiments) Lines represent averages of three replicates. Column graph of slopes of linear regression curves at the 30–60 h time interval (upper graph) and graph of area under curve at the 30–60 h time interval (lower graph). Error bars represent standard deviation of three replicates. Statistic indicators *P ≤ 0.05, **P ≤ 0.01, and ***P ≤ 0.001.

In next step we examined the effects of MpL fusions on the invasion of MCF10A neoT cells, which are a model of aggressive breast cancer cells. In the assay, their invasion relies on effective degradation of Matrigel coating, a gelatinous protein mixture resembling the ECM. Both fusions reduced the invasion of MCF10A neoT cells through Matrigel (Figure 6, invasion graphs). In particular, the CysC-MpL fusion lowered the invasion speed significantly (slopes of linear regression curves) and the cumulative number of invaded cells (area under curve) as compared to control (Figure 6A, right column graphs, Supplementary Figure 8). Its effect were even more pronounced than the effect of intracellular protease inhibitor E64d (Supplementary Figure 8). The use of unlinked CysC and MpL alone resulted in speeds of invasion and cumulative numbers of invaded cells comparable to those of controls, whereas the combination of unlinked CysC and MpL led to a reduced cumulative number of invaded cells, due to lower invasion speed at the beginning of the experiment (Figure 6A, right column graphs, Supplementary Figure 8). Clt-MpL fusion also lowered the invasion speed and the cumulative number of invaded cells (Figure 6B, right column graphs) although the effect was not as pronounced as in the case of CysC-MpL. The combination of unlinked Clt and MpL did not change significantly either the invasion speed or the cumulative number of invaded cells.

## DISCUSSION

**Figure 7:**
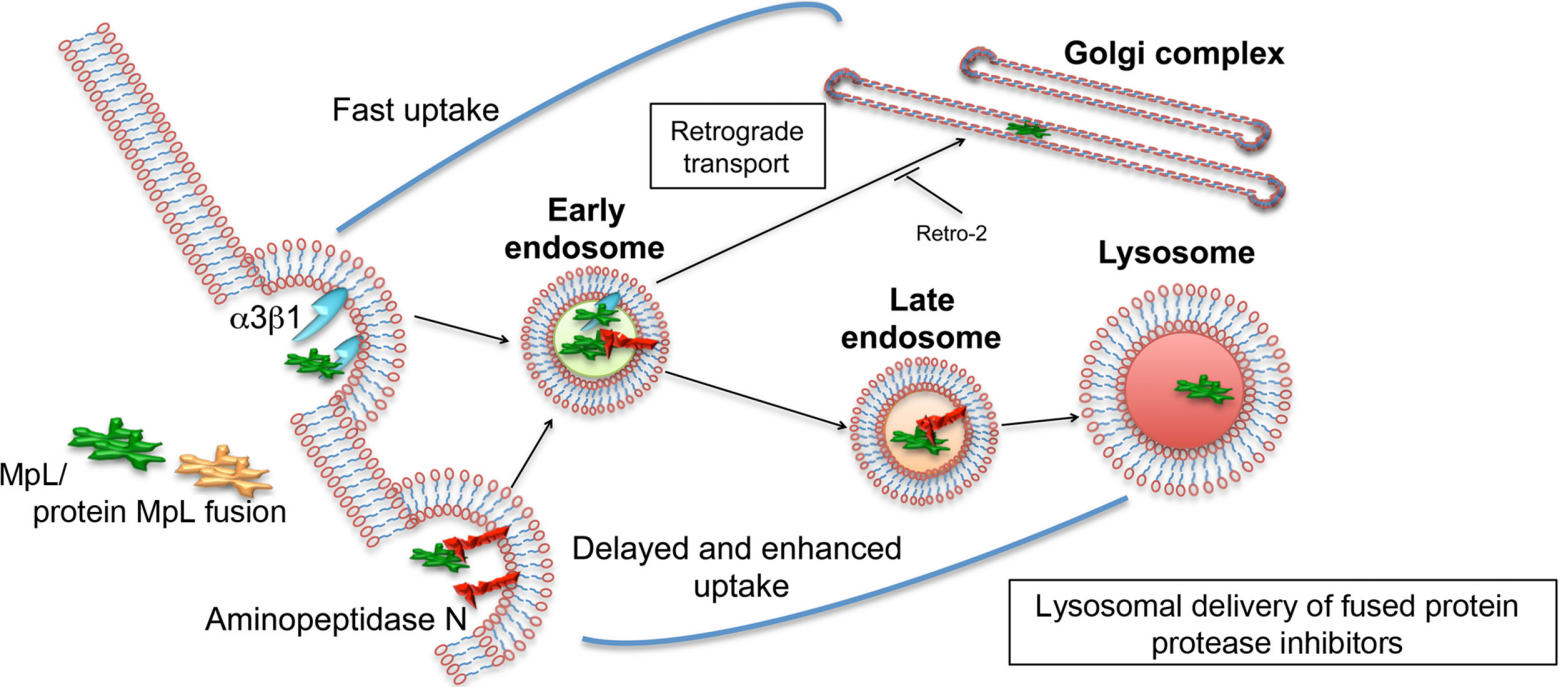
Schematic representation of MpL internalization and subcellular localization in cancer cells MpL can enhance internalization of protein drugs to cancer cells and guide them to lysosomes and/or Golgi apparatus, by binding to aminopeptidase N and/or integrin α3β1.

Lectins are considered as molecules capable of targeted delivery of biological drugs to their intracellular targets, since they specifically bind glycoconjugates on targeted cells and trigger their internalization [29, 30]. In our study we demonstrate that a fungal lectin MpL from edible mushroom *Macrolepiota procera* [7] binds strongly aminopeptidase N/CD13 and α3β1 integrin receptor, glycoproteins that are overexpressed on several types of cancer cells and recognized as candidates for specific cell targeting [31–33]. Through ligand-receptor binding, MpL triggers cell uptake, by clathrin-dependent endocytosis, resulting in its accumulation in GA and endo/lysosomal vesicles (Figure 7). Similar binding and internalization behaviour was also demonstrated for MpL when it was fused with other proteins. The fusion of MpL with cysteine peptidase inhibitors significantly improved their delivery to endo/lysosomal vesicles, resulting in greatly reduced degradation of ECM by their lysosomal targets, cysteine cathepsins, and consequently decreased the migration and invasiveness of cancer cells.

MpL differs from other fungal entomotoxic and nematotoxic β-galactoside-binding lectins, which were shown to have negative effects on the proliferation of mammalian cell lines [6]. Treatment of human cell lines showed that, even at fairly high concentrations, MpL has no effect on cell viability/proliferation, but is rapidly internalized and accumulated within certain subcellular compartments. Internalization of MpL by human cells depends on the functional binding site on the α-repeat, while the nematotoxicity of MpL has been linked to the γ-binding site with unknown carbohydrate binding specificity. A similar nematotoxic β-trefoil type lectin, CNL, exhibits an antiproliferative effect against human cell lines specific for leukemic T cells and dependent on carbohydrate binding on the α-repeat [34]. Furthermore, the β-galactoside binding, actinoporin-type lectins XCL and ABL, the former also highly toxic to insects, showed antiproliferative effects on various mammalian cell lines [35–37].

MpL is rapidly internalized into various human cell lines. Shortly after internalization the majority of MpL co-localized strongly with a signal labelling the components of GA (Figure 7). The same route from early endosomes towards the components of GA, i.e. the trans-Golgi network (TGN) but continuing to the endoplasmic reticulum (ER) has been shown for the plant toxin ricin [8] and the bacterial Shiga toxins [38, 39]. The inhibition of the retrograde transport revealed the existence of another, apparently slower targeting pathway sorting MpL to lysosomal vesicles. Furthermore, co-localisation of MpL with the endo/lysosomal marker LysoTracker, following prolonged incubation in HeLa and MCF10A neoT cells, showed that, once internalized, MpL is retained within the acidic subcellular compartments for at least 24 h hours. Similarly, the β-galactoside-binding lectins XCL and ABL were located at their respective intracellular localization sites after 16 h and 24 h, respectively [37, 40].

Small differences in carbohydrate binding specificity apparently influence the intracellular routing and final destinations of lectins in the cell. MpL, specific for terminal N-acetyllactosamine (Galβ1,4GlcNAc), localizes to GA and the endo/lysosomal system. Similarly, mammalian galectin-1, which binds to long poly-N-acetyllactosamine chains, localizes in the Golgi system but then disappears, without accumulation in lysosomes [41, 42]. Lectin XCL, that binds specifically Galβ1, 3GalNAc-containing glycans localizes to late endosomes and lysosomes, but not to GA [40]. On the other hand, lectin ABL, with apparently the same glycan-binding-specificity as XCL, shows perinuclear accumulation in membrane bound vesicles [37]. Furthermore, the fungal chimerolectin, MOA, specific for Galα1, 3-containing structures, accumulates in late endosomes and does not localize in either of the early endosomes, GA or ER [43]. Despite their different intracellular sorting, fungal lectins MpL, XCL [40], ABL [37] and MOA [43] still exploit clathrin-dependent endocytosis. The latter two, however, also utilize clathrin-independent pathways [37].

MpL is internalized via clathrin-mediated endocytosis and the binding target on the cell membrane probably influences its intracellular sorting and final destination. Clathrin-coated pits can be formed spontaneously and in the absence of ligand, but their stabilization and endocytosis is a ligand/receptor dependent process [44]. We therefore hypothesized that MpL is endocytosed via ligand induced internalization of a glycosylated receptor(s). MpL could mimic a ligand by inducing receptor dimerization leading to internalization of ligand-receptor in clathrin-coated vesicles, or it could bind to lipid raft associated glycolipids and be internalized by the same clathrin-dependent pathway. The latter mechanism of entry has already been shown for Shiga and Cholera toxins [14, 45]. Glycolipids were excluded as binding targets of MpL (not shown) using lectin blot on thin layer chromatography as described [46].

Co-immunoprecipitation and mass spectrometry revealed several potential MpL binding glycoproteins on MCF10A neoT cells, a model cell line of aggressive breast cancer. The strongest target appears to be aminopeptidase N (CD13) which is a 120 kDa glycosylated protein that carries both N- and O-linked glycans.

Aminopeptidase N is a metallopeptidase involved in proteolytic processing of various extracellular proteins, however, independent of its enzymatic activity, aminopeptidase N has been implicated in the regulation of processes such as angiogenesis, antigen presentation, cell-to-cell adhesion and infection with corona viruses [47–49]. The co-localisation of aminopeptidase N and MpL in MCF10A neoT cells was weak at shorter time points (≤ 60 min), however, with prolonged incubation (4 h) the co-localization signal increased within endo/lysosomal ‘dot-like’ structures, suggesting an increase in the internalization of the aminopeptidase N. Aminopeptidase N is normally endocytosed by constitutive/physiological sorting mechanisms [49]. In polarized epithelial cells (HepG2, human liver cancer cells) it was found targeted into distinct sorting endosomes and, eventually, into endo/lysosomes in a clathrin- and dynamin-dependent manner [50]. The dynamics of this process are in accordance with slow endo/lysosomal targeting of MpL. Furthermore, it was shown that the internalization of aminopeptidase N can be triggered by binding of Abs [51], coronavirus, drugs [48], or CD13-targeted vesicles [49]. In contrast, human galectin 3, which specifically binds poly-N-acetyllactosamine (repeating -3Galβ1-4GlcNAcβ1- units) and is another strong aminopeptidase N ligand, failed to induce its internalization [42, 47].

Apart from aminopeptidase N, MpL also bound α3β1 integrin receptor (Figure 7), a protein that is overexpressed in breast cancer cells [32, 33]. Integrins are a family of heterodimeric cell surface receptors mediating adhesion of cells to the proteins of ECM. Binding of MpL to the integrin receptor caused its partial internalization and rapid translocation towards GA-like structures. Integrins have very dynamic structures and are constantly endocytosed from the plasma membrane by formation of clathrin-coated pits, caveolin vesicles or lipid rafts [52]. From early endosomes integrins recycle back to the plasma membrane through endosomes and the perinuclear recycling endosomes or they recycle directly from early endosomes [52]. However, it was recently shown that in HeLa cells endocytosed integrins traffic from recycling endosomes to the TGN and that a component of TGN plays a rate-limiting role in integrin trafficking [52]. Internalization of α3β1 integrin can also be induced with specific Abs [53] as well as by binding to galectin 3 [54]. Therefore, we hypothesize that binding to integrins and recycling through TGN could explain fast internalization and targeting of MpL to components of GA. Fast internalization has been observed also for the lectin MOA that induces internalization and degradation of integrin β1, however it is not clear whether MOA binds integrin β1 directly [43].

Finally, two secreted proteins were detected as MpL targets: fibronectin and zinc-alpha-2 glycoprotein. The former is the extracellular matrix component and the latter the signalling molecule. Even though they appear to be irrelevant to the process of MpL internalization we can hypothesize that MpL could crosslink these components and receptors on the cell surface, potentially leading to their internalization. Furthermore, U937 cell line showed reduced adhesion to fibronectin when treated by MpL at 1.5 μM as determined by a real-time cell analysis (not shown). Nevertheless, the possibility that these two proteins are contaminants from the membrane purification protocol could not be excluded.

The ability of MpL to bind tumor-associated proteins and to induce uptake by cells can be explored to deliver biological drugs to their intracellular targets. This hypothesis has been tested on cysteine cathepsins, lysosomal peptidases, involved in tumor migration, invasion and metastasis. Cysteine cathepsins, the most prominent one in cancer being cathepsin B, are involved in the degradation of the ECM proteins (type IV collagen, laminin and fibronectin), thus facilitating growth and invasion of tumor cells into surrounding tissue and vasculature. After partial extracellular degradation ECM fragments are internalized to tumor cells and completely degraded in lysosomes by cathepsin B and other peptidases [16, 55]. We have demonstrated that low molecular weight inhibitors of cathepsin B, able to inactivate both intra- and extracellular fractions of cathepsin B, effectively impair tumor progression [18]. The same holds for endogenous protein inhibitors cystatins [56], however, their uptake by cells and transport to lysosomes limits their effective inhibition. To enhance the delivery of cystatins to lysosomes therefore, we created fusion proteins of MpL and cystatin C (CysC) and clitocypin (Clt). The fusions followed the same internalization route as unlinked MpL (Supplementary Figure 6) and, moreover, retained strong inhibitory potential towards their proteolytic targets.

We showed that both CysC-MpL and Clt-MpL fusions significantly inhibited Matrigel invasion by MCF10A neoT cells and that the effect of the former was more pronounced, most probably due to the fact that cystatin C is a stronger inhibitor of cathepsin B than clitocypin. Inhibition of migration of MCF10A neoT cells in the absence of Matrigel by CysC-MpL fusion indicated that both cell migration and extracellular matrix degradation is reduced by addition of the fusion protein (Supplementary Figure 9). The stronger effect of CysC-MpL fusion on cell invasion was confirmed by flow cytometry, which showed a significant decrease of DQ-collagen IV degradation only in cells treated with CysC-MpL. As a final point, the partial inhibition of MCF10A neoT invasion upon treatment with the combination of non-fused inhibitor cystatin C and MpL indicated that incubation with lectin can facilitate endocytosis of other molecules present in the medium. Similarly, it has been shown that the fungal lectin XCL stimulates the uptake of poorly internalized molecules via a clathrin-dependent mechanism [40].

## MATERIALS AND METHODS

### Recombinant proteins

Active recombinant fungal lectin MpL and its carbohydrate-binding mutants D22R-MpL (D22R) and D109R-MpL (D109R) were expressed in *Escherichia coli* and purified as described [7].

Active recombinant cystatin C (CysC, GenBank accession number: CR542018) without its signal peptide of 26 amino acids was expressed in *E. coli*. The CysC-coding sequence was amplified using Phusion Hot Start II High-Fidelity DNA Polymerase (Thermo Scientific, Waltham, MA, USA) and primers introducing *NdeI* (rCysC-NdeI-F) and *BamHI* (rCysC-BamHI-R-no fusion) restriction sites (Supplementary Table 1). The fragment was subcloned into an appropriately linearized pET11a expression vector (Novagen, WI, USA). For heterologous expression *E. coli* BL21(DE3) transformed with plasmid pET11a::CysC was cultivated in LB medium supplemented with 100 μg/mL of ampicillin, induced with 1 mM isopropyl β-D-1-thiogalactopyranoside when the OD_600_ reached 0.5 then grown for 6 h at 37°C. Bacterial cells were harvested by centrifugation (8000 × g, 20 min, 4°C; RC-5C Plus, Sorval) and resuspended in 50 mM Tris-HCl, 2 mM EDTA (Serva, Germany), 0.1% Triton X-100 (pH 9), sonicated for 15 min and centrifuged again (12000 × g, 20 min, 4°C). The pellet was suspended in 50 mM Tris-HCl, 2 mM EDTA, 6 M Urea (pH 9) and dialysed against 50 mM Tris-HCl (pH 9). Recombinant CysC was purified using DEAE-Sepharose anion-exchange column equilibrated with the same buffer. Unbound fractions were collected, dialysed against phosphate buffered saline (PBS) and concentrated by ultrafiltration (Amicon UM-3).

Recombinant clitocypin (Clt) was expressed in *E. coli* and purified as described [19].

### Construction, expression and purification of recombinant fusion proteins

Two fusions of peptidase inhibitor and lectin connected by a spacer peptide (GGGGS)_3_ were constructed: Clt-MpL and CysC-MpL. Since N-terminus is essential for inhibitory activity of Cystatin C, N-terminal fusion to MpL was designed for both protease inhibitors. Coding sequences of peptidase inhibitor (Genbank accession numbers: DQ150588 for rClt and CR542018 for CysC) and lectin rMpL (GenBank accession number: HQ449739) were amplified with Phusion Hot Start II High-Fidelity DNA Polymerase (Thermo Scientific) and the corresponding primers (Supplementary Table 1) in a two-step PCR to incorporate restriction sites and sequences that code for parts of the linker peptide. The resulting fragments coding for a peptidase inhibitor and lectin were subcloned into pET22b expression vector (Novagen), yielding fusions linking peptidase inhibitor (Clt or CysC) to lectin MpL. These fusions were then heterologously expressed in *E. coli* BL21(DE3), transformed with the constructed expression vectors as described above for recombinant CysC and were purified as described [7]. As previously published for MpL mutants and fusion proteins dimeric forms were observed [7].

### Functional analysis of recombinant fusions

The functionality of both protein domains of recombinant fusions CysC-MpL and Clt-MpL was analysed. A hemagglutination assay was first performed to determine the activity of the lectin domain, using human blood group B erythrocytes as described [57]. Secondly, the inhibitory activity of CysC-MpL, Clt-MpL and CysC against papain (Sigma, MO, USA) was determined.

The constant of inhibition (*K*_i_) was determined according to Henderson linear equation [58] for tight binding competitive inhibitors. Papain activity (2.6 nM final concentration) following addition of different concentrations of proteins was measured in 0.1 M MES buffer (Sigma), 5 mM DTT (Fermentas, MD, USA), 0.1 mM benzoyloxycarbonyl-Phe-Arg-7-(4-methyl) coumarylamide (Bachem, Switzerland), pH 6.5. The assay was carried out at 25°C and fluorescence measured at excitation/emission wavelengths of 370/460 nm on a microplate reader Infinite M1000 (Tecan Group, Switzerland).

### Viability assay

The viability of different cell lines following addition of protein samples was assessed using the MTS assay CellTiter 96^®^ AQueous One Solution Cell Proliferation Assay (Promega, Madison, WI, USA) according to the manufacturer's instructions. Human cell lines used were: non-differentiated and PMA differentiated histiocytic lymphoma cells (U937: ATCC number CRL-1593.2), malignant non-Hodgkin lymphoma (NK-92: ATCC number CRL-2407), leukemic T cells (Jurkat: ATCC number TIB-152), hepatocellular carcinoma cells (HepG2: ATCC number 59195), neuroblastoma cells (SH-SY5Y; ATCC number CRL-2266), cervical cancer cells (HeLa: ATCC number CCL-2) and Ras-transformed human breast epithelial cell line MCF10A neoT (derived from MCF-10 cell line: ATCC number CRL-10317). For MTS cell viability loss assay seeding number of viable cells per well was 3 × 10^4^ (Jurkat, NK-92 and non-differentiated U937), 0.7 × 10^4^ (HepG2, SH-SY5Y, HeLa and MCF10A neoT) and 6 × 10^4^ PMA differentiated U937. Cells were maintained in the appropriate culture media. Lectin (0.2 μM, 1 μM and 5 μM) was added to the cells in 96-well plates and viability was assessed after 48 h, 72 h and 96 h.

### Immunofluorescence staining

Lectin MpL or MpL fusions (0.63 μM) were added to media of coverslip-attached adherent or to suspension cell lines. At different times after the addition of proteins the coverslip-attached cells were washed with PBS and the suspension cells washed with PBS and centrifuged (Stat Spin Cytofuge, StatSpin, Iris International) onto glass slides. The cells were fixed and permeabilized by 10 min incubation in 4% paraformaldehyde (Electron Microscopy Sciences) in PBS following 10 min incubation in 0.1% Triton X-100 in PBS. For labelling with antibodies against aminopeptidase N (CD13), the cells were fixed for 10 min in ice cold methanol and permeabilized for 5 min in ice cold acetone. Non-specific staining was blocked with 3% BSA in PBS for 1 h. Cells were labelled with primary and secondary antibodies, each time for 1 hour in 3% BSA in PBS. The following primary antibodies were used: affinity-purified rabbit anti-MpL antibody (2 μg/ml, BioGenes, Germany), mouse monoclonal anti-human Golgin-97 antibody (0.5 μg/ml, Life Technologies, CA, USA), mouse monoclonal anti-human CD13 antibody (12.5 μg/ml, R&D Systems, MN, USA), mouse monoclonal anti-human CD29 antibody (12.5 μg/ml, R&D Systems), and mouse monoclonal anti-human CD71 antibody (5 μg/ml, Santa Cruz Biotechnology, USA). The secondary antibodies used were goat anti-rabbit antibodies conjugated with Alexa Fluor 555 (2.5 μg/ml, A-21428), goat anti-rabbit antibodies conjugated with Alexa Fluor 488 (2.5 μg/ml, A-11070) and donkey anti-mouse antibodies conjugated with Alexa Fluor 555 (2.5 μg/ml, A-31570); all were purchased from Life Technologies. Cells were washed three times with PBS after each of the performed steps. The acidic intracellular compartments of cells were stained by 40 min incubation with 60 nM LysoTracker Red DND-99 (Invitrogen- Thermo Scientific) prior to paraformaldehyde fixing. Cells were mounted on slides with Prolong Gold Antifade Reagent containing nuclear 4’,6-diamidino-2-phenylindole (DAPI) stain (Thermo Scientific).

Membranes were labelled with membrane-embedded palmitoylated green fluorescent protein, coded by pCAG-GFP plasmid (Addgene, MA, USA). Transfection of cells was done one day in advance with Lipofectamine 2000 Reagent (Life Technologies), following the manufacturer's instructions.

The inhibition of lectin binding and/or its internalization was achieved by pre-incubation of MpL (0.6 μM) for 30 min at 37°C in growth medium containing lactose (0.1 M final concentration, Sigma), prior to addition to the cells. Chlorpromazine (25 μM final concentration, Sigma) was used for inhibition of clathrin-mediated endocytosis, Filipin III (1,5 μM, Sigma), for inhibition of clathrin-independent caveolar endocytosis and Retro-2 (20 μM, Millipore, Germany) for inhibition of retrograde transport. All inhibitors were added to the cells 1 hour prior to treatment with proteins.

Immunostained cells were visualized with an LSM-710 confocal microscope (Carl Zeiss, Germany) equipped with UV (405 nm), Argon (488 nm and 514 nm) and HeNe (543 nm and 633 nm) lasers. The images were acquired and processed using ZEN software (Carl Zeiss).

### Inhibition of intracellular peptidases

The inhibition of intracellular peptidases upon addition of proteins was measured in MCF10A neoT whole cell lysates. Cells in tissue culture treated 6-well plates were incubated for one and two hours in the presence of 10 μM E64d (Sigma), fusions, MpL, peptidase inhibitor (Clt or CysC) and a combination of unlinked MpL and peptidase inhibitor. All experiments were done in triplicate. The cells were then washed twice in PBS and detached with dissociating reagent TrypLE Select (Gibco, Thermo Scientific). All subsequent steps were carried out at 4°C. Cells were centrifuged (300 × g) and washed twice in PBS. Cell pellets were then resuspended in whole cell lysis buffer [50 mM Hepes, 250 mM NaCl, 0.1% (v/v) NP-40 (Igepal CA-630, Sigma), pH 7] and incubated for 10 minutes. Lysed cells were centrifuged (14000 × g) and the pellet discarded. Protein concentration was determined with DC Protein Assay (Bio-Rad Laboratories, CA, USA) according to the manufacturer's instructions. Cathepsin B and L activity was measured in whole cell lysates (9 μg). Each lysate was analysed in triplicate. Lysates were incubated in black 96-well plates for 10 min before the addition of substrate in 100 mM Hepes, 2 mM EDTA, 5 mM DTT, pH 5 (cathepsin B) or pH 6.5 (cathepsin L). The benzoyloxycarbonyl-Arg-Arg-7-(4-methyl)coumarylamide substrate (20 μM, Bachem) and the benzoyloxycarbonyl-Phe-Arg-7-(4-methyl)coumarylamide substrate (50 μM, Bachem) were added to measure cathepsin B and L activity, respectively. Kinetics were measured (30 min) at 30°C, at excitation/emission wavelengths of 370/460 nm on a microplate reader Infinite M1000 (Tecan). Data were analysed using GraphPad Prism 5 software. Slopes of linear regression curves were calculated and statistical difference was determined using Student's *t*-test.

### Membrane protein isolation, co-immunoprecipitation and mass spectrometry

MCF10A neoT cells were grown in appropriate media and scraped from plates. After two washes in ice cold PBS they were resuspended in fractionation buffer [250 mM sucrose, 20 mM Hepes, 10 mM KCl, 1.5 mM MgCl_2_, 1 mM EDTA (Serva), 1 mM EGTA (Fluka, Germany), 1 mM DTT, pH 7.4] with the protease inhibitor cocktail cOmplete^™^ ULTRA Tablets (Roche, Switzerland). All subsequent steps were carried out at 4°C. Cells were passed through a 27-gauge needle 10 times then lysed in a Dounce homogeniser with 5 pestle strokes. Cell lysate was incubated for 20 min before centrifugation (5 min, 720 × g). The pellet was washed with fresh fractionation buffer, passed through the 27-gauge needle 10 times and centrifuged again (10 min, 720 × g). Two supernatants were combined and centrifuged (20 min, 12000 × g). The resulting supernatant was subjected to centrifugation in a Centrikon T-2070 ultracentrifuge (Kontron Instruments, Germany) in a TST 28.38 rotor for 45 min at 100000 × g. The pellet was resuspended, passed through a 27-gauge needle 10 times and centrifuged again (45 min, 100000 × g). The pellet was dissolved in lysis buffer [50 mM Hepes, 250 mM NaCl, 0.1% (v/v) NP-40 (Igepal CA-630, Sigma), pH 7].

Membrane protein samples (50 μl) were then incubated with MpL (2 μg) in lysis buffer with or without lactose (0.05 M). MpL-target complexes were co-immunoprecipitated with magnetic nanoparticles Dynabeads^®^ Protein G (Invitrogen) according to the manufacturer's instructions. Empty nanoparticles (25 μl) were labelled with 6 μg of affinity purified rabbit anti-MpL (Biogenes) for isolation of MpL complexes. Labelled nanoparticles were added to mixtures of membrane protein samples preincubated with lectin and incubated for 30 min on tube rotator. Nanoparticles were heated for 10 min at 95°C in SDS sample buffer with 20 mM DTT and the proteins resolved on precast 8% Precise^™^ Tris-Glycine gels (Thermo Scientific). Gels were silver stained, individual bands excised and, following in-gel trypsin digestion, identified by mass spectroscopy fingerprinting using an Orbitrap linear trap quadrupole (LTQ) Velos mass spectrometer coupled to a Proxeon nano-LC HPLC unit, (Thermo Fisher Scientific, Waltham, MA, USA). Results were analysed using Scaffold MS software (Proteome Software).

For immunodetection, co-immunoprecipitated MpL-target complexes were resolved on precast 8% Precise^™^ Tris-Glycine gels and transferred (for 4 h at 4°C) onto PVDF membrane. Membranes were blocked for 1 h in 5% non-fat dry milk in tris-buffered saline (TBS) with 0.5% Tween-20. Primary antibodies (anti-human CD13 antibody (2 μg/ml, R&D Systems), and mouse monoclonal anti-human CD29 antibody (1 μg/ml, R&D Systems) diluted in blocking solution were incubated overnight at 4°C. Antibody-reactive proteins were detected by incubation with horseradish peroxidase (HRP)-conjugated secondary anti mouse antibodies (1 : 5000, Jackson ImmunoResearch laboratories. Inc) in blocking solution for 1h. Immunoreactive bands were visualized with LumiLight Plus WesternBlotting substrate (Roche) and images were acquired using a GelDoc System (Bio-Rad).

### The invasion and migration of cells assayed in real time

The invasion and migration of MCF10A neoT cells was assayed in real time on a real-time cell analyser xCELLigence RTCA DP (ACEA Biosciences). For this purpose CIM plates were prepared as described [59]. Lower and upper compartments were filled with different proteins (1.5 μM) or 10 μM E64d. 24 hour serum-starved cells (3 × 10^4^ cells/ well) were added to the upper compartment and the degree of cell invasion or migration measured every 15 minutes for 72 hours. Data were acquired with xCELLigence RTCA Software and analysed with GraphPad Prism 5 software. All experiments were done in triplicate. The cell index was plotted on XY graphs. Slopes of linear regression curves and area under the curve were calculated for specific time intervals. Statistical difference was determined using Student's *t*-test.

### DQ-collagen IV degradation

The effect of fusion proteins and of combinations of proteins on intracellular DQ-collagen IV degradation was assessed according to the protocol described in Mirković et al., [60]. 5 × 10^4^ MCF10A neoT cells were plated into the wells of a 24-well plate. Cells were allowed to attach overnight, when the media were replaced with serum free medium containing 10 μM E64d, 0.63 μM of recombinant fusions or combinations of proteins. After 4 h incubation, DQ-collagen IV (5 μg/ml, Invitrogen) was added and the cells were incubated for an additional 2 h. Propidium iodide (BD Biosciences, CA, USA) exclusion was used to monitor just the viable cells. The measurements were performed on a BD FACS Calibur instrument (Beckton Dickinson, Inc.), analysis using FlowJo software (FlowJO, Ashland, OR, USA).

## CONCLUSIONS

In conclusion, fungal lectin MpL has been shown not to be toxic to human cell lines despite its rapid internalization and sorting to Golgi apparatus and lysosomes, where prolonged retention was observed. For recognition of cell membrane glycoproteins the functional binding site on the α-repeat subunit is needed. The properties of MpL remain preserved when it is fused with other proteins, designating it as a promising carrier of protein drugs to intracellular targets. Facilitated uptake of other molecules by MpL could be of great value for various biomedical and biotechnological applications.

## SUPPLEMENTARY MATERIALS FIGURES AND TABLES


