# Supplementary material for: Fungal lectin MpL enables entry of protein drugs into cancer cells and their subcellular targeting
Source: Oncotarget. 2017 Mar 2;8(16):26896–910. doi: 10.18632/oncotarget.15849 (PMC5432305; doi:10.18632/oncotarget.15849)
Supplement: Supplementary file 1 [file oncotarget-08-26896-s001.pdf]

Fungal lectin MpL enables entry of protein drugs into cancer cells and their subcellular targeting

Supplementary Materials

Supplementary Table 1: List of oligonucleotides used in cloning of recombinant fusion proteins

Recombinant protein	Primer pair	Primer sequence (5′-3′)
Cystatin C	rCysC-NdeI-F	CATATGTCCAGTCCCGGCAAGCCGCCGCGCCTGGT
	rCysC-BamHI-R-no fusion	AAAGGATCCTCATCATCAGGCGTCCTGACAGGTGGAT
Cystatin C as N-terminal part of CysC-MpL fusion	rCysC-NdeI-F	CATATGTCCAGTCCCGGCAAGCCGCCGCGCCTGGT
	rCysC-BamHI-R	GGATCC**GCCTCCACCAGAGCCTCCTCCACC**GGCGTCCTGA CAGGTGGATT
MpL as C-terminal part of MpL fusions	rMpL-BamHI-F	GGATCC**GGAGGCGGAGGGTCG**TCCACCCAAGTCTCCTCTGGCCA
	rMpL-SacI-R	GAGCTCATCATCAAGGACGATCGATTGTC
Clitocypin as N-terminal part of Clt-MpL fusion	rClt-NdeI-F	GGGGCATATGGCTTCTCTTGAGGATGGTAC
	rClt-BamHI-R	GGATCC**GCCTCCACCAGAGCCTCCTCCACC**CTCCCGGGTGAA TCTCCAAG

Introduced restriction enzyme sites are underlined and the introduced linker is indicated in bold type.

Supplementary Table 2: The viability of several human cell lines is unaffected by incubation with MpL for 72 h

	Cell viability			
**Cell line**	**Non-treated cells (viability - %)**	**Treated cells: 0.2 µM (relative viability - %)**	**Treated cells: 1 µM (relative viability - %)**	**Treated cells: 5 µM (relative viability - %)**
HeLa	100.00 ± 2.84	99.23 ± 5.22	101.68 ± 2.17	100.30 ± 4.93
SH-SY5Y	100.00 ± 15.57	136.28 ± 10.62	128.76 ± 2.04	129.71 ± 0.70
HepG2	100.00 ± 3.26	108.29 ± 10.44	109.29 ± 0.92	109.57 ± 4.95
MCF10A neoT	100.00 ± 7.10	99.09 ± 7.48	103.60 ± 5.64	104.95 ± 7.90
U937	100.00 ± 7.02	101.79 ± 3.41	122.13 ± 4.59	129.48 ± 7.71
Differentiated U937	100.00 ± 13.60	119.56 ± 4.74	117.31 ± 2.27	126.15 ± 4.50
NK-92	100.00 ± 1.11	101.81 ± 0.29	112.47 ± 5.54	125.63 ± 3.67
Jurkat	100.00 ± 14.82	136.50 ± 17.31	118.72 ± 1.46	118.45 ± 4.10

Cell viability of different cell lines as measured by the MTS assay shows no significant toxicity of lectin MpL at concentrations from 0.2 to 5 µM.

Supplementary Table 3: The viability of several human cell lines is unaffected by incubation with MpL for 96 h

	Cell viability			
**Cell line**	**Non-treated cells (viability - %)**	**Treated cells: 0.2 µM (relative viability - %)**	**Treated cells: 1 µM (relative viability - %)**	**Treated cells: 5 µM (relative viability - %)**
HeLa	100.00 ± 1.79	100.05 ± 14.21	100.05 ± 3.91	100.05 ± 1.24
SH-SY5Y	100.00 ± 1.82	103.59 ± 9.33	102.57 ± 2.17	104.93 ± 1.75
HepG2	100.00 ± 8.05	105.37 ± 9.48	105.37 ± 7.14	105.37 ± 2.11
MCF10A neoT	100.00 ± 0.70	91.56 ± 8.24	97.05 ± 1.28	102.22 ± 1.15
U937	100.00 ± 3.58	80.44 ± 20.40	102.94 ± 7.13	101.16 ± 8.37
Differentiated U937	100.00 ± 2.55	87.91 ± 20.53	97.94 ± 4.67	106.69 ± 2.31
NK-92	100.00 ± 12.91	82.91 ± 11.24	101.24 ± 5.17	128.83 ± 1.95
Jurkat	100.00 ± 10.99	103.63 ± 8.33	106.30 ± 3.55	93.59 ± 17.84

Cell viability of different cell lines as measured by the MTS assay shows no significant toxicity of lectin MpL at concentrations from 0.2 to 5 µM.

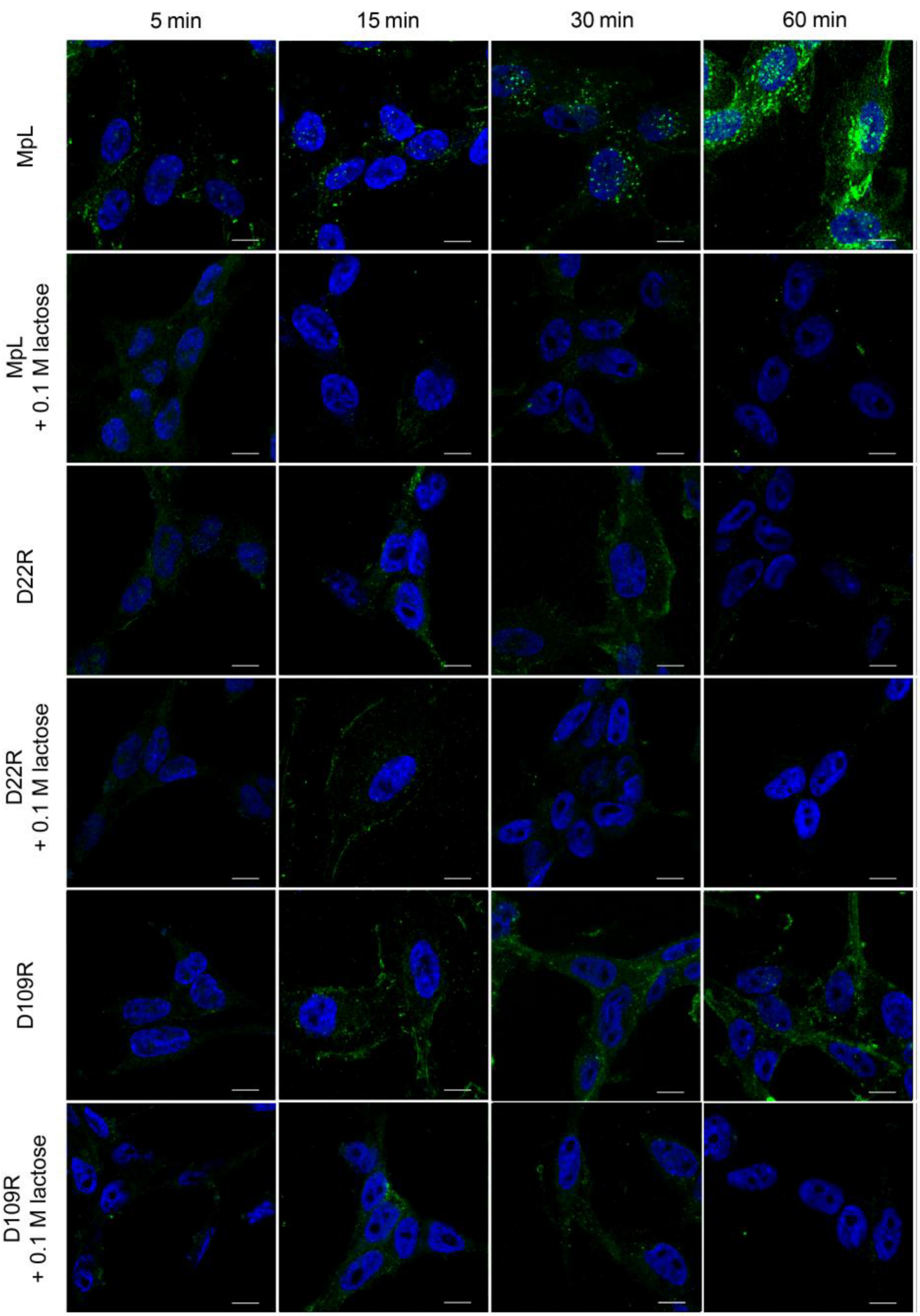

Supplementary Figure 1: Localization of MpL and non-glycan-binding MpL mutants in MCF10A neoT cells. Localization of MpL, D22R and D109R in MCF10A neoT cells, in the absence or presence of 0.1 M lactose at 5, 15, 30 and 60 min after addition of lectins. Cells were stained with anti-MpL antibodies and secondary goat anti-rabbit antibodies conjugated with Alexa Fluor 488. Images were taken at 63x magnification. Bar, 10 µm.

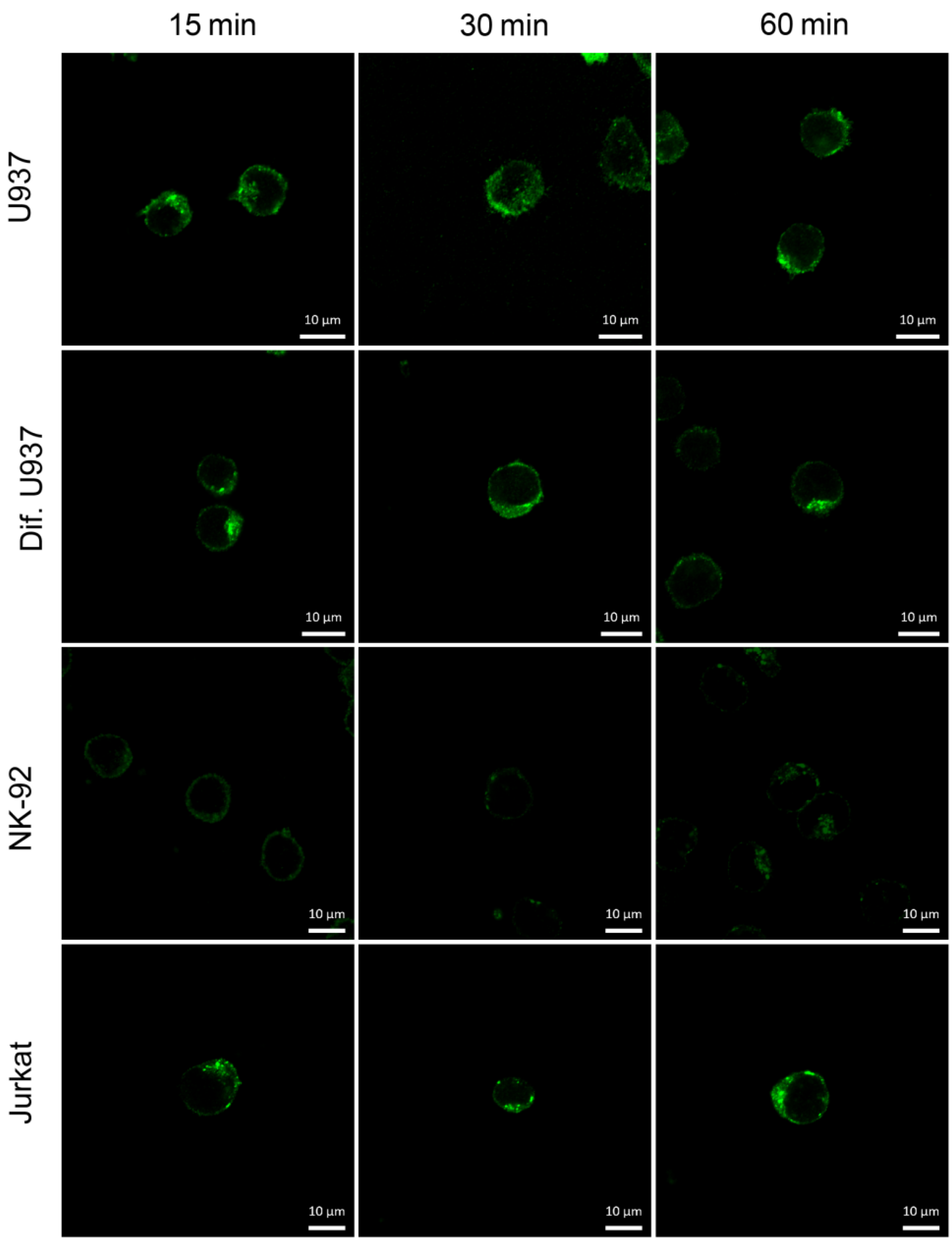

Supplementary Figure 2: Localization of MpL in different cells. Localization of MpL in non-differentiated and differentiated U937 cells, NK-92 cells and Jurkat cells at 15, 30 and 60 min after addition of MpL. Cells were stained with rabbit polyclonal anti-MpL antibodies and secondary goat anti-rabbit antibodies conjugated with Alexa Fluor 488. Images were taken at 63x magnification. Binding of MpL mutant forms is not shown.

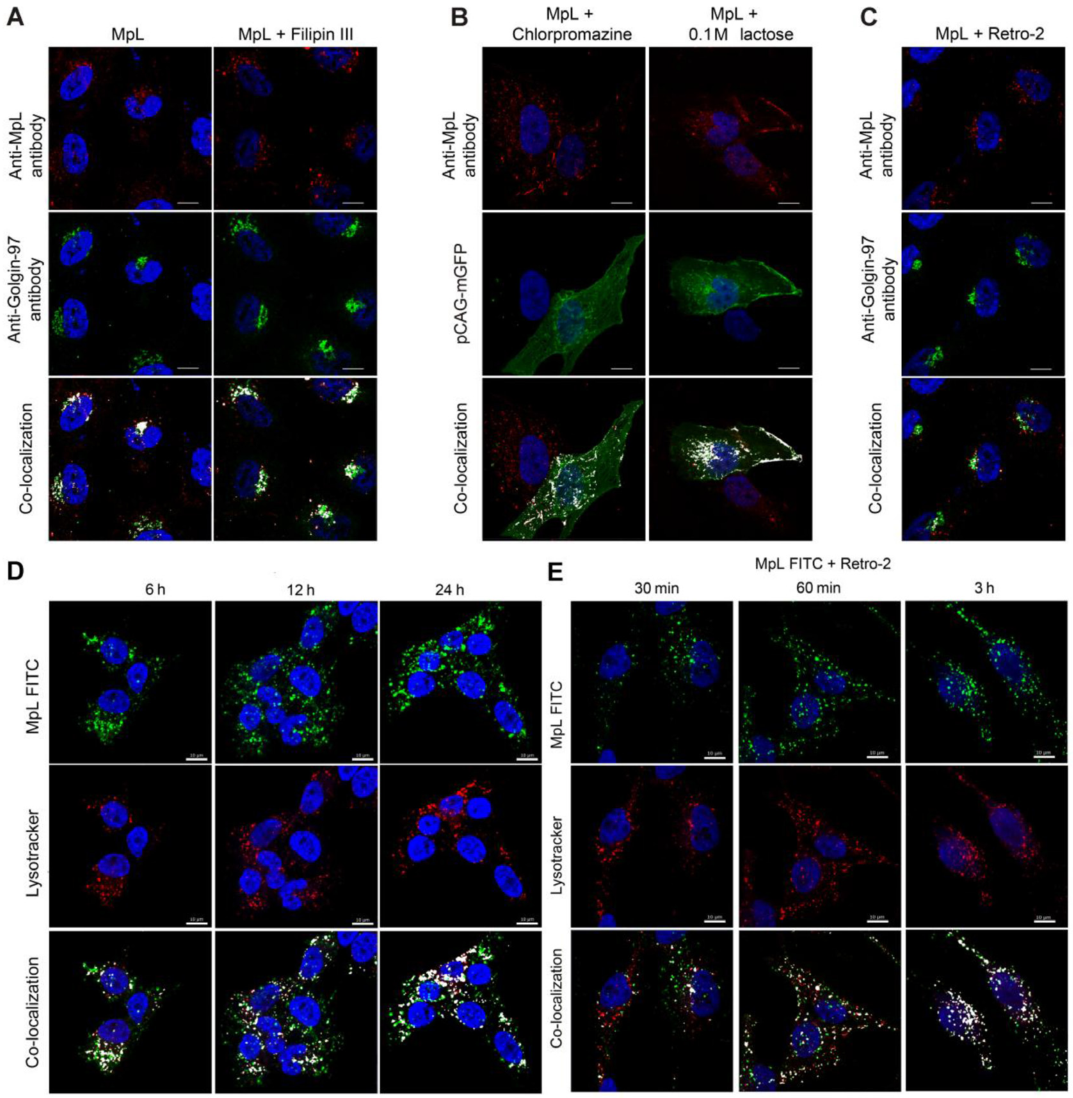

Supplementary Figure 3: Mechanisms of MpL cell uptake and sorting in MCF10A neoT cells. (**A**) Localization of MpL in MCF10A neoT cells after 3 h incubation in the presence or absence of Filipin III. Cells were double labelled with anti-MpL antibodies and anti-human Golgin-97 antibodies and secondary antibodies conjugated with Alexa Fluor 488 (MpL) and Alexa Fluor 555 (Golgin-97). (**B**) Localization of MpL on the plasma membrane of MCF10A neoT cells after 3 h incubation in the presence of chlorpromazine or 0.1 M lactose. Cells were transfected with the pCAG-mGFP plasmid for membrane staining and stained with anti-MpL antibodies and secondary antibodies conjugated with Alexa Fluor 555. (**C**) Localization of MpL in MCF10A neoT cells after 3 h incubation in the presence of Retro-2. Cells were double labelled with anti-MpL antibodies and anti-human Golgin-97 antibodies and secondary antibodies conjugated with Alexa Fluor 488 (MpL) and Alexa Fluor 555 (Golgin-97). (**D**) Localization of FITC labelled MpL in MCF10A neoT cells after 6 h, 12 h and 24 h incubation (**E**) Localization of FITC labelled MpL in MCF10A neoT cells after 30 min, 60 min and 3 h in the presence of Retro-2. LysoTracker Red DND-99 was added to cell cultures 40 min prior to paraformaldehyde fixation. Images were taken at 63x magnification. Co-localization is shown in the merged pictures with white pixels.

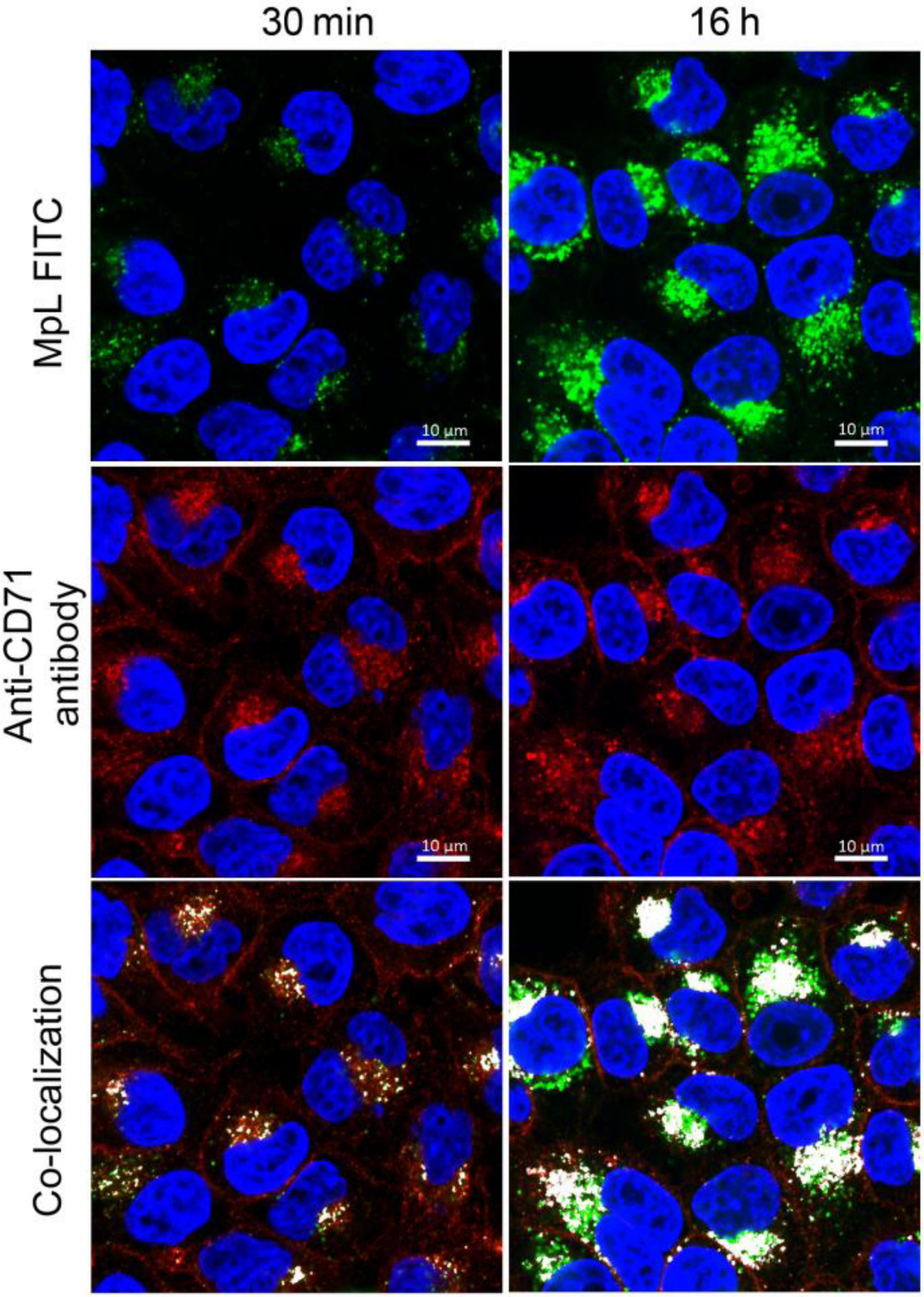

Supplementary Figure 4: FITC labelled MpL co-localizes with transferrin receptor (CD71) in HeLa cells. Localization of FITC labelled MpL in HeLa cells after 30 min and 16 h incubation. Cells were stained with mouse monoclonal anti-CD71 antibodies and secondary donkey anti-mouse antibodies conjugated with Alexa Fluor 555. Images were taken at 63× magnification. Co-localization is shown in the merged pictures with white pixels.

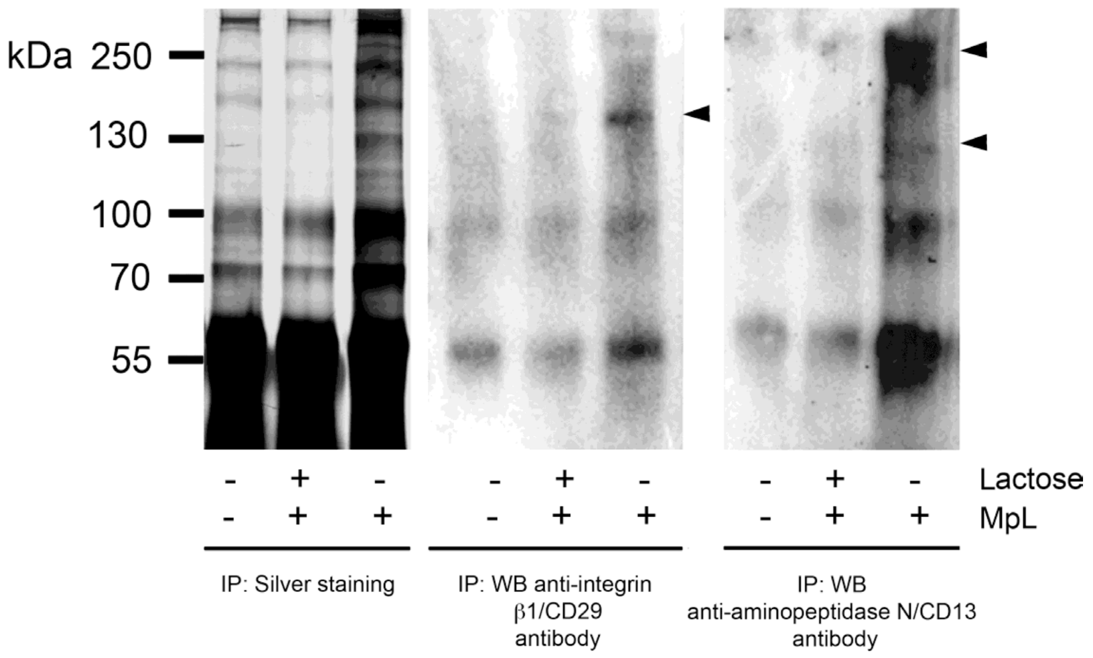

Supplementary Figure 5: Immunoprecipitation of MpL binding membrane proteins of MCF10A neoT cells: (Left) Silver staining of membrane proteins precipitated with rabbit anti-MpL antibody after incubation with PBS (Left lane), lactose and MpL (Middle lane) and MpL (Right lane). Western blot analysis for the (Middle) integrin β1/CD29 and (Right) aminopeptidase N/CD13 immunoreactivity indicated by arrows of MpL binding membrane proteins precipitated with rabbit anti-MpL antibody after incubation with PBS (Left lane), lactose and MpL (Middle lane) and MpL (Right lane).

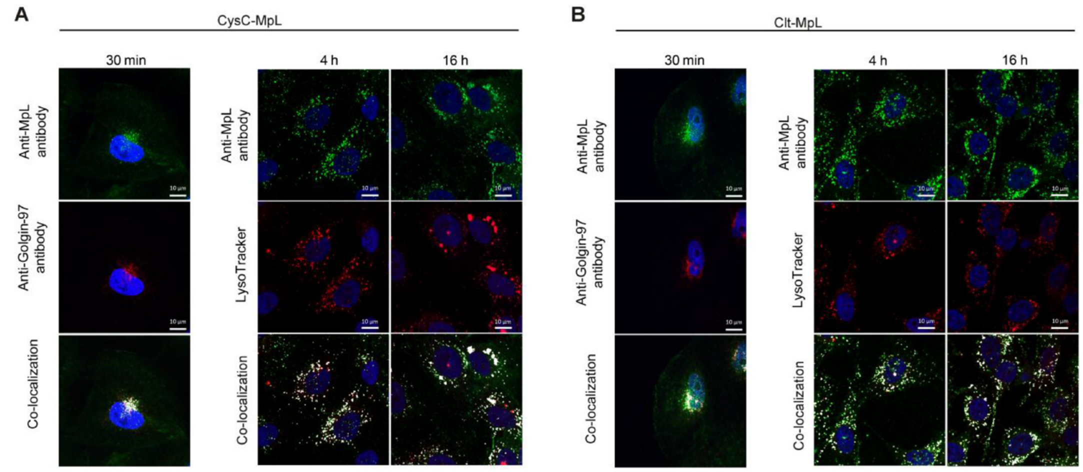

Supplementary Figure 6: MpL fusion proteins follow the same internalization pathway as the unlinked MpL protein in MCF10A neoT cells. Left panel – Co-localization of CysC-MpL (**A**) and Clt-MpL (**B**) fusion protein with a marker of the Golgi apparatus (Golgin-97) 30 min after its addition. Cells were double labelled with anti-MpL antibodies and anti-human Golgi-97 antibodies and secondary antibodies conjugated with Alexa Fluor 488 (MpL) and Alexa Fluor 555 (Golgin-97). Right panel –Co-localization of CysC-MpL (A) and Clt-MpL (B) fusion protein with a marker of endo/lysosomes (LysoTracker Red DND-99) 4 h and 16 h after addition. LysoTracker Red DND-99 was added to cell cultures 40 min prior to paraformaldehyde fixation. Images were taken at 63x magnification. Co-localization is shown in the merged pictures with white pixels.

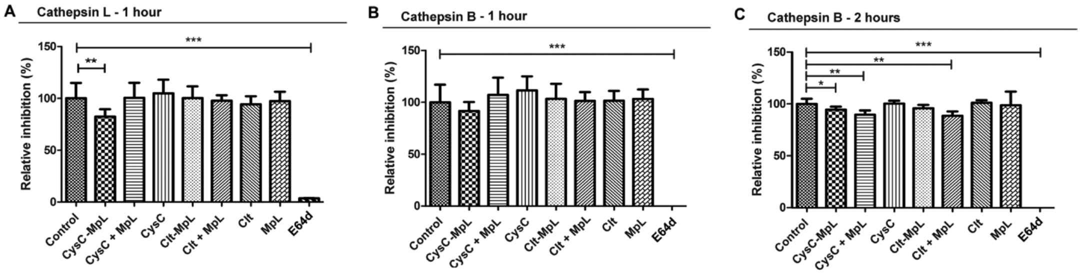

Supplementary Figure 7: Inhibition of cathepsin B and L in MCF10A neoT whole cell lysates by fusion proteins CysC-MpL and Clt-MpL. Peptidase activity was measured in whole cell lysates prepared one or two hours after the addition of proteins or E-64d. (**A**) Activity of cathepsin L after one hour incubation. (**B**) Activity of cathepsin B after one hour of incubation. (**C**) Activity of cathepsin B after two hour incubation. Error bars represent standard deviation of three replicates. Statistic indicators: $^{*}P \leq 0.05$, $^{**}P \leq 0.01$ and $^{***}P \leq 0.001$.

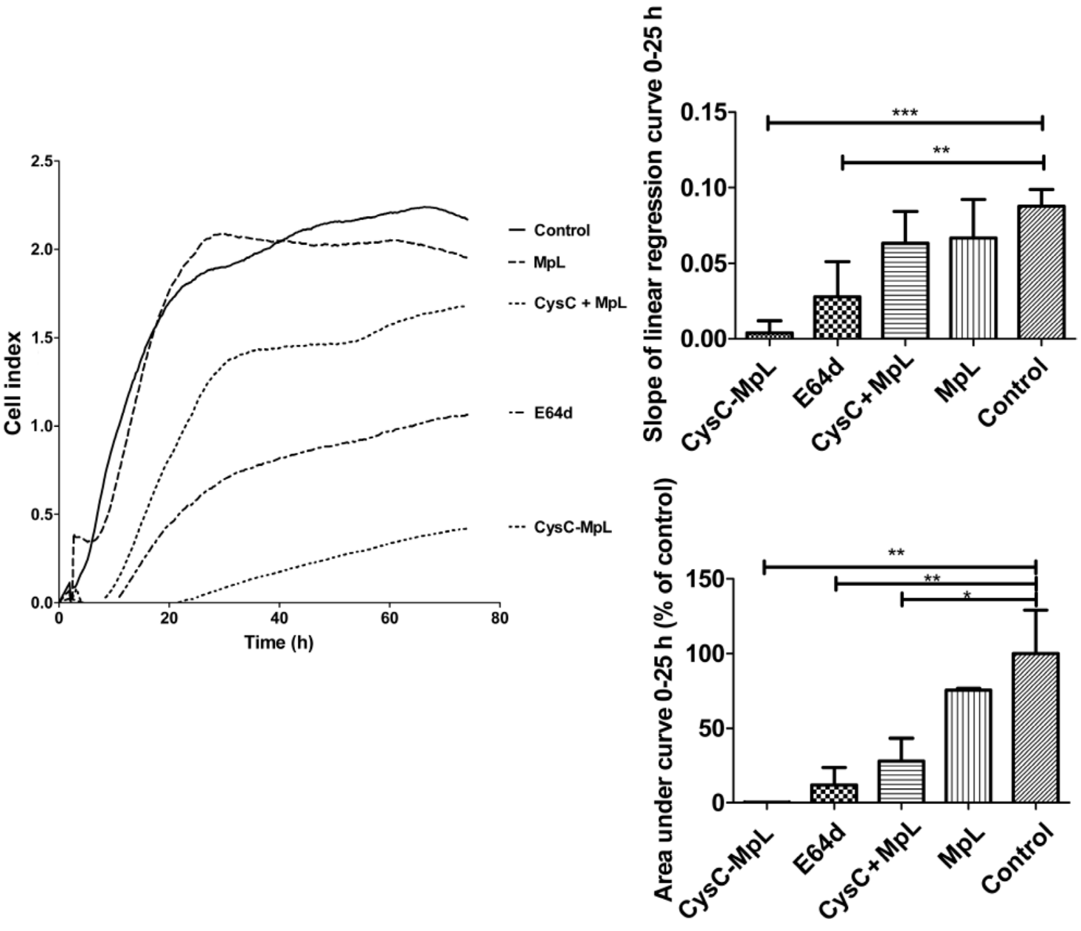

Supplementary Figure 8: Inhibition of invasion of MCF10A neoT breast cancer cells through Matrigel coating by fusion protein CysC-MpL. Invasion of serum-starved MCF10A neoT cells was measured on a real-time cell analyser xCELLigence using CIM plates and Matrigel in the 72 hour time period. Lines represent averages of three replicates. Column graph of slopes of linear regression curves at the 0–25 h time interval (upper graph) and graph of area under curve at the 0–25 h time interval (lower graph). Error bars represent standard deviation of three replicates. Statistic indicators $^{*}P \leq 0.05$, $^{**}P \leq 0.01$, and $^{***}P \leq 0.001$.

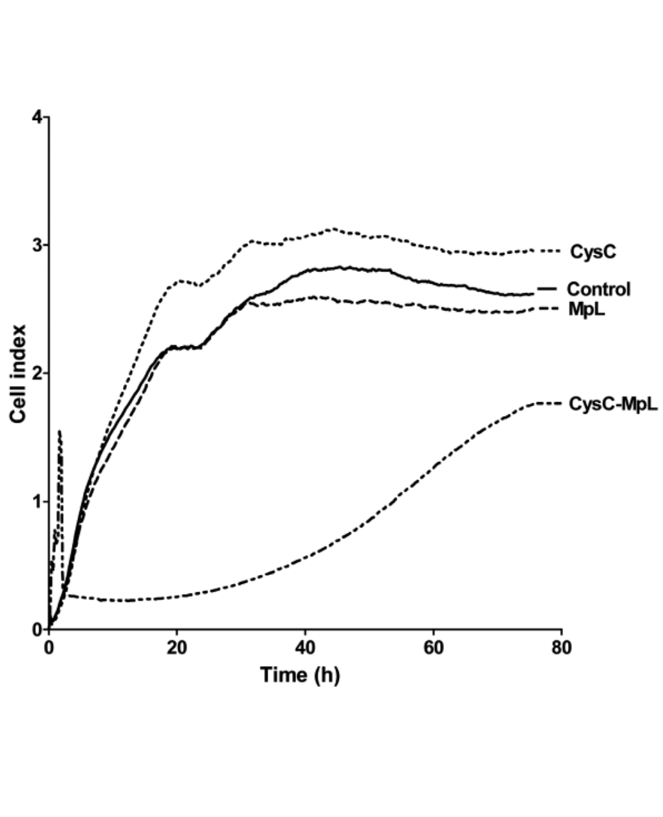

Supplementary Figure 9: Inhibition of migration of MCF10A neoT breast cancer cells by fusion protein CysC-MpL. Invasion of serum-starved MCF10A neoT cells was measured on a real-time cell analyser xCELLigence using fibronectin coated CIM plates in the 72 hour time period. Lines represent averages of three replicates. Column graph of slopes of linear regression curves at the 0–20 h time interval (upper graph) and graph of area under curve at the 0–20 h time interval (lower graph). Error bars represent standard deviation of three replicates. Statistic indicators $^{**}P \leq 0.01$ and $^{***}P \leq 0.001$.
